# Group-Based Interventions for Carers of People With Dementia: A Systematic Review

**DOI:** 10.1093/geroni/igac011

**Published:** 2022-03-06

**Authors:** Bethany McLoughlin

**Affiliations:** Warwick Medical School, University of Warwick, Coventry, United Kingdom

**Keywords:** Caregivers, Social Support, Well-being

## Abstract

**Background and Objectives:**

It is well documented that caring for someone with dementia is associated with many negative mental health outcomes, such as depression, anxiety, and a reduction in quality of life. Group-based interventions are one strategy for improving well-being for carers, but previous systematic reviews have reported inconsistent findings about the efficacy of group-based interventions for carers of people with dementia.

**Research Design and Methods:**

This systematic review investigates the qualitative and quantitative evidence for the effectiveness of group-based interventions and identifies targets for future research. Narrative synthesis was used to analyze the data.

**Results:**

A comprehensive search of 4 databases revealed 117 potentially relevant studies, 19 of which met the full inclusion criteria. Five studies investigated group cognitive behavioral therapy, 8 investigated psycho-educational interventions, and 6 investigated support groups. The effectiveness of the interventions varied widely, even within subcategories. No type of intervention was consistently shown to improve well-being, though qualitative data and data about participant satisfaction was generally very positive.

**Discussion and Implications:**

Based on the quality and quantity of the evidence currently available, there is not enough evidence to reach firm conclusions about the impact of group-based interventions on well-being. In order to establish the effectiveness of group-based interventions there needs to be more high-quality studies with larger sample sizes about this topic. Future research may benefit from the use of mixed methods data collection to explore the disparity between qualitative and quantitative findings in the literature.

**Translational Significance**

Systematic reviews investigating group-based interventions for carers of people with dementia have reported inconsistent findings about their efficacy. The present work aimed to investigate the factors driving these mixed findings and to identify targets for future research. The review identified a disparity between the findings of the qualitative studies (highly positive) and quantitative studies (mixed), which could suggest that future research may benefit from using a mixed methods design to explore this discrepancy. The review also highlighted the need for more high-quality research to be conducted on this topic that includes larger sample sizes and longer-term interventions.

## Background

It is estimated that 50 million people have dementia globally, with this figure predicted to triple by 2050 (World Health Organisation, 2017). This is leading to an increasing burden on informal carers as well as health and social care providers (Prince et al., 2016). The contribution of informal carers is essential for meeting the needs of people with dementia, with informal carers of people with dementia providing an estimated 82 billion hours of care worldwide every year (Wimo et al., 2018).

Caring for someone with dementia is associated with many negative mental health outcomes, such as depression, anxiety, and a reduction in quality of life (Cooper et al., 2007; Farina et al., 2017; Sörensen et al., 2006). Given the growing number of informal carers of people with dementia (Lewis et al., 2014; World Health Organisation, 2017) and the impact of caring on some carers’ mental health, it is vital to find ways to effectively support carers.

## Support for Carers

One possible avenue to support carers is through group-based interventions. They have the advantage that multiple people can participate at the same time, which may make them more cost-effective than individually tailored interventions (McDermut et al., 2001; Shapiro et al., 1982) as well as creating an opportunity to receive social support and advice from peers (Leung et al., 2015).

However, evidence of their effectiveness for carers of people with dementia is limited. Recent systematic reviews regarding psycho-social interventions for carers have reported inconsistent findings (Cheng and Zhang, 2020; Dam et al., 2016; Dickinson et al., 2017; Hopkinson et al., 2019), with many concluding that the effectiveness of group-based interventions varies widely between studies and does not appear to consistently improve well-being. A weakness of these reviews is that they do not focus on group-based interventions exclusively, and so authors have grouped all group-based intervention studies in the same category regardless of content, characteristics, and target population. With such heterogeneity in the studies included, it is unsurprising that no clear pattern of findings emerges.

Currently, there are few reviews that have focused solely on group-based interventions. One such review conducted by Toseland and Rossiter (1989) found no evidence of improvement to carer burden, stress, or any other standardized measure of well-being after attending a carer support group intervention. However, in studies included in the review that distributed satisfaction and evaluation questionnaires, carers consistently reported highly positive evaluations of the interventions and high levels of satisfaction. In a more recent review of quantitative research, Chien et al. (2011) found that group-based interventions led to a moderate improvement to carer mental health and depression and a small improvement to carer burden, but acknowledged that there was a large degree of heterogeneity between the effectiveness of the included interventions. Chien et al. compared three types of group-based interventions (mutual support group, psycho-educational group, and educational group) and found that psycho-educational groups were the most effective at improving carer well-being, while educational groups were more effective at improving carer burden. A review of qualitative research into support groups for carers of people with dementia conducted by Lauritzen et al. (2015) found carers consistently reported that support groups had a positive impact on their well-being and ability to cope with their caregiving situation.

## Review Aims

The aim of the present work is to conduct a systematic review of qualitative, quantitative, and mixed methods evidence to (a) investigate the effectiveness of group-based interventions at improving the well-being of informal carers, (b) identify future targets for research, and (c) make recommendations about best practice for providing support groups considering the quality of evidence available. By focusing exclusively on group-based interventions, we sought to investigate the different types of group-based interventions separately from each other in order to make sense of the inconsistencies described in previous reviews, and to identify how the type of group-based intervention and the environmental and research context within which it is delivered may influence its effectiveness. The present work is the first to include qualitative, quantitative, and mixed methods research around group-based interventions for carers of people with dementia. This is valuable because it allows for the exploration of the disparate findings between the qualitative and quantitative reviews which were discussed previously. Additionally, the present work is able to capture more up-to-date research and fill gaps in knowledge highlighted by previous reviews.

## Methods

### Search Strategy

A comprehensive search was conducted of the MEDLINE, PsycINFO, EMBASE, and Web of Science databases to examine the evidence of the effectiveness of group-based interventions for carers of people with dementia. Search terms included all known synonyms, truncations, and MeSH terms where possible and appropriate, to ensure that the search was extensive. Search strategies were kept as similar as possible across databases (Online Supplementary Material Section A for search strategies). The search was conducted June 5, 2020.

### Inclusion and Exclusion Criteria

Inclusion and exclusion criteria were developed within a PICOS (Population, Intervention, Comparison, Outcomes, and Study design) framework, which is a tool used to develop research questions for systematic reviews (Liberati et al., 2009). Studies were included if they met the following criteria: (a) the intervention was aimed at carers of people with any type of dementia, (b) the intervention was group-based, and (c) outcomes included any measure of well-being/carer-reported experiences about the intervention. No restrictions were put on study design, study location, or publication date.

One reviewer appraised studies, and studies were excluded if: (a) the intervention also included carers of people with other conditions, (b) the groups in the intervention were made up exclusively of members of a single-family (i.e., family therapy), (c) the study solely investigated intervention satisfaction with no measure of well-being, (d) it was a pilot study, or (e) the study was found to be of very low quality using critical appraisal checklists and significant issues with their research design or methods were identified (details on critical appraisal are provided in the “quality assessment” section below). Additional exclusion criteria included: (a) research not written in English, (b) case reports, literature reviews, books, and discussion articles, and (c) multi-component interventions if the group-based component was not the main focus as it would not be possible to establish the direct contribution of the group-based component to changes in well-being.

### Selection Procedure and Data Extraction

All titles and abstracts were assessed for eligibility, and irrelevant studies were discarded. The full text was then accessed and assessed against inclusion/exclusion criteria. The following data from studies that met the requirements for inclusion was extracted and entered into a predesigned table: (a) author(s) and location, (b) publication year, (c) journal of publication, (d) study design, (e) type of intervention, (f) characteristics of intervention, (g) participant demographics, (h) type of data collected, (i) key findings, and (j) quality of evidence. Data extraction tables can be found in the Supplementary Material for this article (Online Supplementary Material Section B).

### Quality Assessment

The quality of included studies was assessed using standardized Critical Appraisal Skills Programme (CASP) checklists for qualitative and randomized control trial (RCT) design studies (CASP, 2017, 2018). The Joanna Briggs Institute (JBI) (2016) Critical Appraisal checklist for quasi-experimental studies were used for nonrandomised quantitative studies and the Mixed-Methods Appraisal Tool (MMAT) checklist was used to evaluate mixed methods research (Hong et al., 2018).

Research that was found to be of low quality and/or at high risk of bias based on these checklists was excluded. This occurred when the study failed to meet the initial quality screening criteria (e.g., for MMAT “1. Are there clear research questions?” and “2. Do the collected data allow to address the research questions?” [Hong et al., 2018]), or the study failed to meet the majority of quality criteria on the full appraisal checklist which led to the identification of major problems with the study design or methods. Studies that were included in the review were classified as low, moderate, or high-quality studies based on the proportion of the checklist criteria the study met and the severity of problems identified.

### Data Synthesis

This review took a narrative approach to synthesis following the guidelines of Popay et al. (2006), which consists of four stages: (a) developing a theory of how the intervention works, why, and for whom, (b) developing a preliminary synthesis of findings of included studies, (c) exploring relationships in the data, and (d) assessing the robustness of the synthesis. No meta-analysis was conducted as the review included both quantitative and qualitative studies, and as there was a large range of outcome measures used in the studies included in this review, a meta-analysis would add little statistical value.

Studies were categorized by type of intervention and grouped into tables (group-based cognitive behavioral therapy [CBT] interventions, group-based psycho-educational interventions, and support groups), and findings within each subcategory are summarized in a narrative description in the results section. These subcategories were not predetermined but were created based on the studies that passed the screening process for inclusion in the review. This means that not all types of group-based intervention are represented in this review if no research that met the inclusion criteria was found in the literature search.

## Results

**Figure 1.**
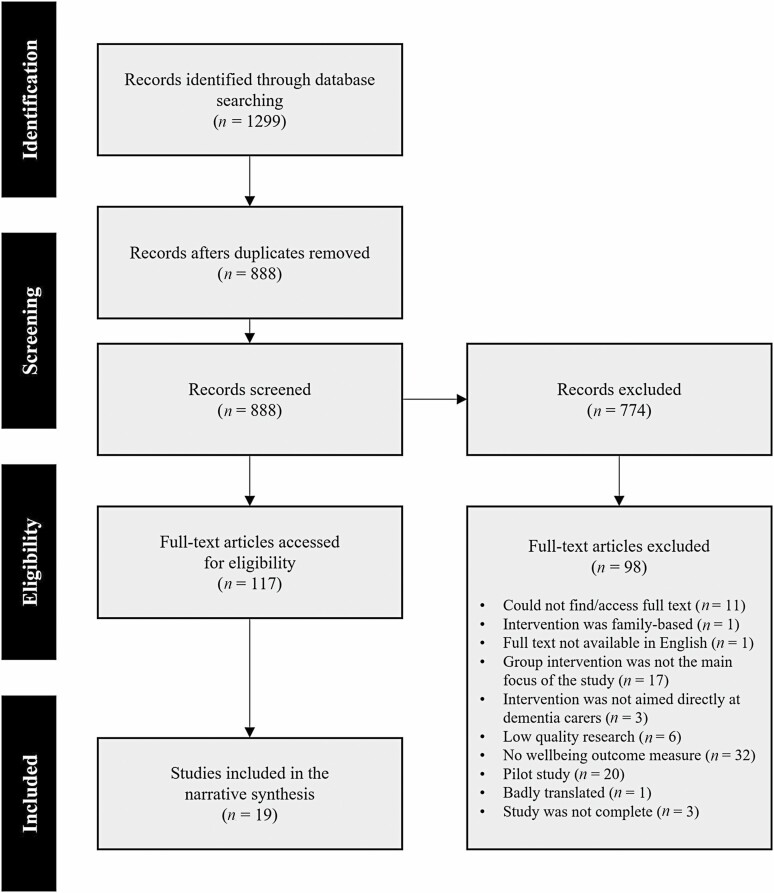
Preferred Reporting Items for Systematic Reviews and Meta-Analyses (PRISMA) flowchart illustrating the inclusion of studies in this review.

A total of 1,299 records were captured from the database searches, and 888 records remained after duplicates were removed. Based on screening titles and abstracts, 117 papers that met the criteria for full-text screening were identified. The full text of 117 papers were assessed and a total of 19 eligible reviews were identified as fitting the inclusion criteria and quality requirements of this review. Reasons for exclusion at the full-text screening stage can be found in Figure 1.

A total of 19 studies were included in this review. Of those included, five studies were group CBT interventions, six were support group interventions, and eight were psycho-educational interventions. Fifteen studies were quantitative, three were mixed methods, and one was qualitative. Table 1 summarizes the study characteristics of the studies included in each subcategory.

**Table 1.** Characteristics of the Studies Included in Each Subcategory

	Subcategory		
Characteristics	Group CBT (5 studies)	Psycho-educational (8 studies)	Support group (6 studies)
*Study characteristics*			
Research type	5 Quantitative (4 RCTs, 1 quasi-experimental)	2 Mixed methods 6 Quantitative (3 RCTs, 1 quasi-experimental, 4 pretest–posttest)	1 Qualitative 1 Mixed methods 4 Quantitative (3 RCTs, 1 quasi-experimental, 1 pretest–posttest)
Location	2 Europe 2 North America 1 South America	5 Europe 1 North America 2 Asia	2 Europe 2 North America 2 Asia
Number of participants			
*M*	64.2	166.1	50.3
Range	35–102	19–308	25–103
Study completion rate, *M*	71.2%^a^	74.3%^a^	80.4%
Control group	Psycho-educational support group (4 studies) Usual care/self-help manual (1 study)	Usual care (4 studies) N/A (4 studies)	Usual care (4 studies) N/A (2 studies)
Research quality	Low (2 studies) Moderate (3 studies)	Low (1 study) Moderate (7 studies)	Low (1 study) Moderate (5 studies)
*Intervention characteristics*			
Number of sessions			
*M*	7	7.9	36^a^
Range	5–8	4–15	12–104
Session length	90 min (3 studies) 2 h (2 studies)	90 min (3 studies) 2 h (4 studies) 8 h (1 study)	1 h (5 studies) Not reported (1 study)
Size of group	5–10 people^a^	5–11 people^a^	6–7 people^a^
Delivery method	100% face-to-face	100% face-to-face	5 face-to-face 1 telephone
*Participant characteristics*			
Age	Mean: 60.6 y	Mean: 59.7 y^a^	Mean: 63.46 y^a^
Gender	Mean: 73.7% female	Mean: 76.6% female^a^	Mean: 83.7% female^a^
Relationship with care recipient			
Spouse	51.83%^a^	47%^a^	56%^a^
Parent	32.83%^a^	45%^a^	33%^a^
Other	15.33%^a^	9%^a^	9%^a^
Type of dementia of care recipient	2 Alzheimer’s disease 3 Any	1 Alzheimer’s disease 7 Any	2 Alzheimer’s disease or vascular dementia 4 Any

*Note:* CBT = cognitive behavioral therapy; RCT = randomised control trial.

^a^Indicates that not all studies in the subcategory reported this data, please refer to Online Supplementary Material Section B for data extraction tables.

### Group CBT Interventions

Five studies (Aboulafia-Brakha et al., 2014; Arango-Lasprilla et al., 2014; Gendron et al., 1996; Gonyea et al., 2006; Passoni et al., 2014) adapted CBT for delivery in a group format. Their content was similar overall, although the emphasis on certain topics varied slightly between the interventions. All included CBT techniques for avoiding negative thoughts, advice for understanding and managing the symptoms of dementia, and information about self-care. Three studies also included some degree of assertiveness training to empower carers to discuss the division of caring responsibilities with other family members and/or to request the support they need from healthcare services (Online Supplementary Material Section C presents a summary of intervention content to highlight similarities and differences in content).

### Effectiveness of Group CBT Interventions

A wide variety of measures were used across the studies in this subcategory (Table 2 for a summary of measures used within each study and the study findings). It should be noted that some measures were only used in one study, and therefore conclusions about the effectiveness of group CBT interventions at improving that aspect of well-being should be made with additional caution.

**Table 2.** Summary of the Findings of the Five Studies Included in the Group CBT Interventions Subcategory

Measure	Aboulafia-Brakha et al. (2014)	Arango Lasprilla et al. (2009)	Gendron et al. (1996)	Gonyea et al. (2006)	Passoni et al. (2014)
Salivary cortisol	Yes	Not measured	Not measured	Not measured	Not measured
Stress	No	No	Not measured	Not measured	Not measured
Burden	No	Yes	No	No	Not measured
Depression	No	Yes	No	Not measured	No
Anxiety	No	Not measured	No	Not measured	No
Life satisfaction	Not measured	Yes	Not measured	Not measured	Not measured
Negative thinking	Not measured	Not measured	No	Not measured	Not measured
Assertiveness	Not measured	Not measured	Yes	Not measured	Not measured
Distress	Not measured	Not measured	Not measured	Yes	Not measured
Carer needs	Not measured	Not measured	Not measured	Not measured	Yes

*Notes:* Cells containing “No” indicate that the intervention did not have a significant effect on that measure and cells containing “Yes” indicate that the intervention did have a significant effect on that measure. CBT = cognitive behavioral therapy.

No significant improvement to stress, anxiety, and negative thinking in carers of people with dementia was found in any study, which is unexpected as this is the main goal of CBT. Findings about burden and depression were inconsistent, with only the study conducted by Arango-Lasprilla et al. (2009) finding an improvement to these measures. This study also found a significant improvement to life satisfaction in the intervention group. There do not seem to be any major differences in the content of the study conducted by Arango-Lasprilla et al. (2014) that could explain why that intervention was found to be more effective. However, the participants in this study were made up of carers who were caring for their parents or other family members, rather than spousal carers which were more common in other studies. This difference in participant characteristics is likely due to societal expectations about caring responsibilities in Colombia, and these cultural differences could also be driving the findings about effectiveness. It is possible that group CBT is less effective for spousal carers or that interventions should tailor their content to specific types of caring situations in order to maximize their effectiveness, rather than applying a one-size-fits-all approach. Although assertiveness training was a part of three interventions (Online Supplementary Material Section C for descriptions of the content of the interventions), only the study conducted by Gendron et al. (1996) used assertiveness as an outcome measure. This study found a significant improvement to assertiveness postintervention, but there were no significant improvements for any other outcome measure. This is at odds with the satisfaction questionnaire completed by participants after the intervention, with all participants indicating they were “satisfied” or “very satisfied” with the intervention, and 97.1% of participants reporting that it “helped them cope with their situation more effectively.”

Aboulafia-Brakha et al. (2014) saw a reduction in salivatory cortisol levels in the CBT group, which would suggest a reduction in stress levels, but saw no improvement in any other measure, including stress. Gonyea et al. (2006) found a reduction in distress in response to neuropsychiatric symptoms in the care recipient, but did not find a reduction in the participants’ overall level of distress or burden. This specific benefit may stem from the intervention in that study being shorter (only five sessions compared to eight) and the content of the study being more targeted towards managing and understanding challenging behavior associated with dementia. This finding could suggest that CBT interventions could be more successful if they focus on one specific topic. Finally, one study (Passoni et al., 2014) found an improvement in “carer needs,” which was a measure of the level of support the carer was receiving. This finding simply meant that carers were accessing more support (e.g., respite care) at the end of the intervention than the beginning of the intervention. This does not necessarily mean that the content of the intervention improved the carer’s quality of life and ability to cope, but it could suggest that signposting to other support during the intervention may have been beneficial.

### Group-based Psycho-educational Interventions

Interventions were classified as psycho-educational if the aim of the intervention was to provide participants with information and/or training about dementia and caring. For example, this training could include advice about developing problem-solving skills or techniques to manage difficult symptoms that are common in dementia.

There was some variation in the content included in the interventions in this subcategory (Online Supplementary Material Section D for a summary of intervention content). All interventions provided participants with information about managing challenging symptoms in dementia and general information about dementia to some degree. Four studies provided advice about planning for the future, how to access support, and self-care. Two studies provided advice about managing depression and information about medication for the care recipient. Lewis et al. (2009) was the only study that concentrated on educating participants about different relaxation techniques, which means that the results of that study should not necessarily be grouped with the other studies as the focus was very different.

There was noteworthy variation between the studies in this subcategory in how many sessions the intervention consisted of and how long these sessions were. For example, the study conducted by Hsu et al. (2017) consisted of only four sessions, but the sessions were 8-h long, which was highly irregular compared to the rest of the studies where sessions were 90–120 min long. This could have led to the high rate of participants dropping out of the intervention (35.8%), as the authors believe that participants found the intervention too intensive and could not focus for that length of time. Conversely, the intervention with the greatest number of sessions was Pihet and Kipfer (2018) which consisted of 15 2-h sessions which had an attendance rate of 92%. The interventions have similar levels of commitment in terms of hours attending the intervention (32 h for the Hsu et al. study and 30 h for the Pihet & Kipfer study), but the high rate of study completion and attendance seen in the Pihet and Kipfer study may indicate that shorter sessions completed over a longer period of time may be more manageable for carers to attend. However, as both of these studies only had a small number of participants, this claim should be considered with caution.

### Effectiveness of Group-based Psycho-educational Interventions

There was a wide variety of measures used in the studies, with most measures only being used in one or two studies (Table 3 for a summary of study measures and findings).

**Table 3.** Summary of the Findings of the Eight Studies Included in the Group-based Psycho-educational Interventions Subcategory

Measure	Signe and Elmståhl (2008)	Hsu et al. (2017)	Küçükgüçlü et al. (2018)	Kurz et al. (2010)	Lewis et al. (2009)	Martín-Carrasco et al. (2014)	Pihet and Kipfer (2018)	Ulstein et al. (2007)
Burden	Unclear^a^	Yes	Yes	Not measured	Yes	No	Yes	Not measured
Life satisfaction	Yes	Not measured	Not measured	Not measured	Not measured	Not measured	Not measured	Not measured
Health	Not measured	Unclear^b^	Not measured	Not measured	Not measured	Unclear^c^	Not measured	Not measured
Quality of life	Not measured	Not measured	Not measured	No	Yes	No	Not measured	Not measured
Depression	Not measured	Not measured	Not measured	No	Yes	Not measured	Not measured	Not measured
Stress	Not measured	Not measured	Not measured	Not measured	Yes	Not measured	Not measured	No
Anxiety	Not measured	Not measured	Not measured	Not measured	Yes	Not measured	Not measured	Not measured
Anger	Not measured	Not measured	Not measured	Not measured	Yes	Not measured	Not measured	Not measured
Distress	Not measured	Not measured	Not measured	Not measured	Not measured	No	Unclear^d^	Not measured
Self-efficacy	Not measured	Not measured	Not measured	Not measured	Not measured	Not measured	Yes	Not measured

*Notes:* Cells containing “No” indicate that the intervention did not have a significant positive effect on that measure, the cells containing “Unclear” indicate that there may have been an improvement for specific items of the questionnaire but no significant improvement overall (the specific improvement is explained in this caption below) and cells containing “Yes” indicate that the intervention did have a significant positive effect on that measure.

^a^The intervention did not lead to a statistically significant decrease in burden compared to the control group, but the intervention group had a significant delay for nursing home placement for the care recipient compared to the control group.

^b^There was no statistically significant improvement to the physical health of the participants but there was an improvement on the mental health aspects of the questionnaire.

^c^There was a statistically significant decrease in anxiety and insomnia items on the health measure in the intervention group postintervention. However, there was no significant improvement in any other measure.

^d^Psychological distress improved significantly but distress in response to the psychiatric symptoms of the care recipient did not improve postintervention.

Burden was used as a measure in six of the eight studies, and the findings appear to suggest that the interventions led to a decrease in burden. However, two of the studies that found a significant change to burden had very small sample sizes, and one study focused mainly on relaxation techniques rather than providing educational content about dementia and caring. Therefore, further high-quality research is necessary before strong conclusions about the impact of group-based psycho-educational interventions on burden can be made. The study by Lewis et al. (2009) that focused mainly on relaxation found positive results in all outcome measures, and this could suggest that relaxation techniques may improve well-being in carers of people with dementia. This could be a potential target for future research.

As the remaining nine measures were only used in 1–3 studies each (Table 3) and generally indicated mixed levels of effectiveness, this does not warrant further analysis as there is not enough evidence to reach conclusions about the impact of psycho-educational interventions on those aspects of well-being. However, it should be noted that studies that included a measure of participant satisfaction or qualitative data collection techniques found highly favorable reviews of the intervention, which clashes with the mixed and statistically insignificant findings about well-being. This again raises the concern that standardized measures of well-being are not capturing the experiences of the participants.

Three studies (Kurz et al., 2010; Martín-Carrasco et al., 2014; Ulstein et al., 2007) failed to find any positive impact of their interventions on well-being and concluded that the intervention was no better than usual care. Notably, the intervention in the study conducted by Martín-Carrasco et al. was developed based off a similar educational intervention that had been delivered in an individual format with positive results (Martín‐Carrasco et al., 2009), but these positive outcomes did not translate to a group setting. All three sets of authors concluded that this is because educational interventions may not be appropriate for group settings, as the advice included in the intervention may not be relevant for every participant’s situation, and that this sort of intervention should be delivered on an individual basis or tailored to a specific group of carers (e.g., female spousal carers or care recipients with similar symptoms and dementia severity).

### Support Group Interventions

Interventions were classified as support groups if the intervention consisted of a small group of carers who gathered to talk through their problems and find social support. The focus of interventions in this subcategory was the exchange and discussion of experiences rather than following a set manual of education or type of therapy. However, two studies had some predetermined psycho-educational topics of discussion as talking points (e.g., advice about improving communication techniques).

Six outcome measures (quality of life, stress, burden, depression, distress, and personal gains) were used across the five studies that used quantitative data collection techniques (Table 4 for summary of study measures and findings).

**Table 4.** Summary of the Findings of the Five Studies That Used Quantitative Data Collection Techniques Included in the Support Group Interventions Subcategory

Measure	Acton and Miller (1996)	Berger et al. (2004)	Chu et al. (2011)	Fung and Chien (2002)	Winter and Gitlin (2007)
Quality of life	No	Not measured	Not measured	Yes	Not measured
Stress	No	Not measured	Not measured	Not measured	Not measured
Burden	Not measured	No	No	Not measured	No
Depression	Not measured	No	No	Not measured	No
Distress	Not measured	Not measured	Not measured	Yes	Not measured
Personal gains	Not measured	Not measured	Not measured	Not measured	No

*Note:* Cells containing “No” indicate that the intervention did not have a significant effect on that measure and cells containing “Yes” indicate that the intervention did have a significant effect on that measure.

Four of the studies found no positive effects in any outcome measure postintervention. Though one study (Fung and Chien, 2002) did find positive effects (improvement to quality of life and reduction in distress), it should be acknowledged that this study had a relatively small sample size, and the effects could be culturally specific. This information should be taken into account before generalizing the findings to make recommendations about practice elsewhere.

Two studies in this subcategory collected qualitative data through interviews. The themes identified by both studies (Acton and Miller, 1996; Lauritzen et al., 2019) strongly suggest that support groups have a positive effect on the well-being of the carers in these studies (Table 5). Both studies emphasized the importance of interaction with others in finding a sense of acceptance and self-esteem. The findings of Lauritzen et al. also suggest that support groups can improve carer’s understanding of the ethics of caregiving, which was valued by the carers in the study. Other interventions have not reported this, which could be a limitation of using standardized measures as they can only capture a set amount of information and participants cannot contribute information regarding what they found valuable about their experiences outside of the fixed questions on the questionnaire.

**Table 5.** Main Themes Found by the Two Studies That Used Qualitative Data Collection Techniques Included in the Support Group Interventions Subcategory

Study	Theme	Definition
Acton and Miller (1996)	Affiliation	Feeling connected to others
	Individuation	Improvement to the sense of self and gaining a sense of control
	Self-acceptance	Feeling better about themselves
	Healing	Gaining a feeling of peace
Lauritzen et al. (2019)	Emotional well‐being due to peer and family support	Positive interactions with others lifted the carer’s moods and allowed them to express their emotions and hear other people’s perspectives on similar problems
	Emotional sense of togetherness despite hardships	Connecting with others in similar situations raised carers self-esteem and confidence
	Emotional and ethical considerations in caregiving	Gaining an understanding of how to treat the caregiver with respect and dignity as their condition progresses and ensure that they receive the same treatment from others

The positive findings of the qualitative aspects of the studies are at odds with the quantitative findings, even within the same study in the case of Acton and Miller (1996). It is unclear why participants are self-reporting improvements to well-being in an interview but are not self-reporting improvements to well-being in questionnaires. It could be the case that the standardized measures are not asking the right questions, or options on a Likert scale are not sensitive enough to detect small changes in well-being. It is also possible that participants feel a social pressure to give positive feedback about the intervention. Ultimately, based on the small amount of evidence available it is unclear why there is a mismatch between these two data types, and this justifies the need for further, and ideally, mixed methods research to investigate this phenomenon.

## Discussion

This review was the first to report exclusively on group-based interventions for carers of people with dementia that included qualitative, quantitative, and mixed methods research. The review synthesized findings regarding three subcategories of interventions across 19 studies.

### Effectiveness of Group-Based Interventions

#### Group CBT interventions

Overall, this review found no strong evidence to suggest that group CBT interventions are an effective way to improve well-being in carers of people with dementia. However, the research included in this subcategory is not of sufficient size or quality to justify generalizing this conclusion to the entire population of carers, and group CBT may be an appropriate treatment choice for some carers. More research would need to be carried out to investigate the hypothesis that CBT interventions that have been tailored more towards a specific population (e.g., spousal carers, people who care for their parent.) or specific aspect of well-being could be more effective.

#### Group psycho-educational interventions

As findings about the effectiveness of psycho-educational interventions at improving well-being were so mixed between the studies included in this review, it is not possible to draw firm conclusions about the effectiveness of interventions of this type. It is somewhat surprising that no positive effects were found in the psycho-educational control groups for the group CBT interventions discussed previously, as some of the interventions in the group psycho-educational subcategory were quite successful. Ultimately, this highlights the need for more research to be conducted in this area.

#### Support group interventions

Qualitative data about support group interventions seemed to indicate that the interventions were well received and improved participants’ well-being. This was at odds with the quantitative findings, which suggested that the interventions were not effective. This discrepancy between qualitative and quantitative data even occurred within the same study in the case of Acton and Miller (1996). It is unclear why participants self-reported improvements to well-being in a qualitative interview but did not self-report improvements to well-being in quantitative questionnaires. It could be the case that the standardized measures are not asking the right questions, or the multiple-choice options are not sensitive enough to detect small changes in well-being. It is also possible that participants felt a social pressure to give positive feedback about the intervention. Ultimately, based on the small amount of evidence available it is unclear why there is a mismatch between these two data types, and this justifies the need for further, and ideally, mixed methods research to investigate this phenomenon.

#### General discussion

Findings in all subcategories were extremely mixed and there is not enough evidence to draw conclusions about the impact of group-based interventions on the well-being of carers of people with dementia. Although quantitative data from the studies included in this review suggested that group-based interventions are generally ineffective, qualitative data and data about participant satisfaction was generally very positive. This disparity between qualitative and quantitative findings brings into question whether the interventions really did not help carers, if standardized measures are not appropriate for capturing carer well-being in this context, or if the most applicable measures were selected. For example, qualitative data seems to suggest that one of the main benefits of group-based interventions is finding social support and connection, but none of the quantitative studies in this review included a measure of social support or social isolation.

It should be noted that 15 of the studies included in this review were quantitative, three were mixed methods, and only one was qualitative. This indicates that there is a gap in literature for research including qualitative research methods as this could be more appropriate for investigating the experiences of carers and explaining the mixed findings in the literature.

### Comparison of Intervention Types

This review found no compelling evidence to suggest that psycho-educational support groups are the most effective type of intervention at improving well-being (Chien et al., 2011), though there was limited evidence that psycho-educational support groups may lead to a reduction carer burden.

Overall, there was insufficient evidence of positive effects in any type of intervention, so it would not be appropriate to make recommendations for best practice based on the strength of this evidence. Ultimately, there do not seem to be enough studies of sufficiently high quality in each subcategory to justify an in-depth comparison. However, this review has identified multiple targets for future research.

### Research Quality and Limitations

The quality of the research included in this review ranged from low to moderate, however, this was largely driven by difficulties that are unavoidable in this particular population. This review revealed several difficulties with research involving carers of people with dementia, and possible explanations for the lack of improvement seen in quantitative measures of well-being will be discussed in this section.

Firstly, participant attrition and low attendance at interventions was an issue, due to participants having many responsibilities and also the chance of the care recipient passing away or becoming seriously ill. Additionally, due to the time commitment and energy required to participate in an intervention, it is possible that carers who have comparatively better mental health and greater access to resources are more likely to be able to complete the intervention. Not only does this mean that the participants in these interventions might not be representative of the general population and the carers in most need of support, but this also means that they are not as likely to show an improvement in well-being as they were already coping with their situation well to begin with. One way of ensuring that research is accessible for vulnerable carers to participate in is to provide respite care whilst the carer attends the intervention, which could be an option for future research.

Another difficulty when researching this population is that it is unethical to prevent carers from accessing other support, and it may be necessary to provide some type of carer support to those in the control group rather than treatment as usual. It is likely that if a participant is assigned to a control group that receives no intervention, they may seek support elsewhere. As both groups are receiving help, this may lessen the difference between the intervention group and the control group, and this could be one reason why many studies in this review failed to find any significant improvements in the intervention group compared to the control group. Ultimately, there is no ethical way around this issue, and it is a general limitation of all research about this population.

Another confounding variable that results from research with this population is that support networks are likely to vary widely between individuals (i.e., some participants will have no support outside of the study’s intervention, whilst other participants may have professional carers to assist them several times a day). Due to these strong cofounding variables, a well-designed RCT on this topic would require a very large sample size to counteract the noise caused by the large degree of heterogeneity in the circumstances of participants. Therefore, it may be beneficial for future research to utilize qualitative data collection techniques to explore a participant’s experiences with an intervention or to implement a measure of the level of support a carer has to explore if this is associated with the impact an intervention has on well-being.

An alternative explanation for the findings of this review is that the progressive nature of dementia is the reason positive effects are not seen in carers in the intervention group. The burden of caring does not go away as the care recipient’s needs will only grow over time, so perhaps it is unsurprising that carers do not show a reduction in burden. Perhaps instead, the lack of difference in participant responses preintervention and postintervention could suggest that the intervention is acting as more of a buffer or protective measure against loss of well-being, preventing conditions from worsening. For this reason, it is possible that finding a statistically significant improvement on standardized measures is not likely, and qualitative data collection techniques such as interviews may be more useful for understanding the complex emotional state and coping strategies of carers.

It is also possible that the interventions included in the review are not long enough. The prognosis of dementia is variable, and it is possible that someone with dementia will need many years of care. In this time period, as dementia is progressive, their needs and symptoms will be constantly evolving and the information their carer learned in an 8-week intervention several months ago will quickly become outdated. This highlights the need for high-quality longitudinal studies to investigate the impact of longer-term group-based interventions that will evolve over time to be relevant as the dementia progresses.

Finally, another possible limitation of the research included in this review that could explain the lack of positive results is that the majority of the studies included in the review recruited carers of people with any type of dementia. There are many different types of dementia, and the presentation of symptoms varies widely. If the interventions are too general or are just aimed at carers of people with Alzheimer’s disease, this could mean that many carers are not receiving relevant advice. If a significant proportion of carers are not getting the right advice, this could explain why statistically significant improvements are not seen in the group overall. There is a clear need for interventions that are more flexible and specifically tailored to certain types of carers.

## Review Limitations

An important consideration that must be made when interpreting this review is that cultural differences between the research countries could mean that the findings may not be generalizable to other populations or appropriate for data synthesis. It was clear from the large differences in carer demographics between countries, especially concerning gender and relationship to the care recipient, that there are prominent differences between cultures when it comes to societal expectations of carers. Additionally, due to different socio-economic situations and healthcare systems, the participants in these studies had drastically different access to support services based on what country the research was conducted in, which could have a strong impact on well-being. In order to make recommendations about best practice, ideally the evidence behind the recommendation should be generalizable to the population at hand which would involve limiting the research in the review to one country or region. However, the literature search revealed very little research that has been conducted on this topic overall, so there would not have been enough studies to justify limiting the inclusion criteria to one country/region as this would have added little value. More research needs to be done to investigate support groups for carers of people with dementia, and once there is a broader literature a culture-specific review could be conducted which could make better recommendations about best practice.

### Future Directions

A potential target for future research is social activity groups for carers of people with dementia. Currently, there are many social activities available to carers, such as dementia cafés, singing groups, ballroom dancing groups, and so on (Capus, 2005; Camic et al., 2013; Skinner et al., 2018). These types of interventions are focused on reducing social isolation and having fun. The present review did not identify any appropriate studies about social activity groups that met the inclusion criteria, which suggests that there is a gap in the literature for high-quality research focusing on interventions of this type and their impact on the well-being of carers.

Similarly, there were no studies investigating online-based interventions for carers of people with dementia that met the criteria to be included in this review. As technology advances, online interventions are becoming increasingly viable and are seen as a potential solution to overcoming barriers for attending support groups, such as poor mobility or lack of access to transportation and respite care. Due to the COVID-19 pandemic which has been ongoing during the present work, many groups for carers of people with dementia have moved online, which has created an opportunity for the effectiveness of online groups to be studied.

## Conclusion

There is limited quantitative evidence to suggest that group-based interventions for carers of people with dementia are effective. However, as participants generally indicate high satisfaction with the interventions that they took part in, this raises the possibility that qualitative and mixed methods studies may be more appropriate methods of investigating this topic in the future, especially as there was very little existing qualitative and mixed methods research identified by this study. There is also a need for high-quality studies with large sample sizes investigating longer interventions, interventions that target a specific audience, and different types of intervention that were not included in this review, such as online interventions.

## Supplementary Material

igac011_suppl_Supplementary_Material
