# Supplementary material for: Group-Based Interventions for Carers of People With Dementia: A Systematic Review
Source: Innov Aging. 2022 Mar 6;6(3):igac011. doi: 10.1093/geroni/igac011 (PMC9074812; doi:10.1093/geroni/igac011)
Supplement: igac011_suppl_Supplementary_Material [file igac011_suppl_supplementary_material.docx]

**Online Supplementary Material Section** **A**

*Full database search strategy.*

**Medline Search Strategy**

| 1. carer.mp. or exp Caregivers/  **OR**  2. ("informal care*" or "famil* care*" or "spous* care*" or "unpaid care*").mp. [mp=title, abstract, original title, name of substance word, subject heading word, floating sub-heading word, keyword heading word, organism supplementary concept word, protocol supplementary concept word, rare disease supplementary concept word, unique identifier, synonyms] |
| --- |
| **AND** |
| 3. support group.mp. or exp Self-Help Groups/  **OR**  4. therapy group.mp. or exp Psychotherapy, Group/  **OR**  5. group counseling.mp.  **OR**  6. ("group based" adj5 intervention).mp. [mp=title, abstract, original title, name of substance word, subject heading word, floating sub-heading word, keyword heading word, organism supplementary concept word, protocol supplementary concept word, rare disease supplementary concept word, unique identifier, synonyms]  **OR**  7. ("group based" adj5 program*).mp. [mp=title, abstract, original title, name of substance word, subject heading word, floating sub-heading word, keyword heading word, organism supplementary concept word, protocol supplementary concept word, rare disease supplementary concept word, unique identifier, synonyms] |
| **AND** |
| 8. dementia.mp. or exp Dementia/ or exp Frontotemporal Dementia/ or exp Dementia, Vascular/ or exp Dementia, Multi-Infarct/  **OR**  9. Alzheimer's disease.mp. or exp Alzheimer Disease/ |
| **AND** |
| 10. Limit to English Language |

**Embase Search Strategy**

| 1. carer.mp. or exp caregiver/  **OR**  2. ("informal care*" or "famil* care*" or "spous* care*" or "unpaid care*").mp. [mp=title, abstract, heading word, drug trade name, original title, device manufacturer, drug manufacturer, device trade name, keyword, floating subheading word, candidate term word] |
| --- |
| **AND** |
| 3. support group.mp. or exp support group/ or exp self help/  **OR**  4. exp group therapy/ or therapy group.mp.  **OR**  5. group counseling.mp.  **OR**  6. ("group based" adj4 intervention).mp. [mp=title, abstract, heading word, drug trade name, original title, device manufacturer, drug manufacturer, device trade name, keyword, floating subheading word, candidate term word]  **OR**  7. ("group based" adj4 program*).mp. [mp=title, abstract, heading word, drug trade name, original title, device manufacturer, drug manufacturer, device trade name, keyword, floating subheading word, candidate term word] |
| **AND** |
| 8. exp semantic dementia/ or exp dementia/ or exp senile dementia/ or exp multiinfarct dementia/ or exp frontal variant frontotemporal dementia/ or exp presenile dementia/ or dementia.mp. or exp frontotemporal dementia/  **OR**  9. alzheimer's disease.mp. or exp Alzheimer disease/ |
| **AND** |
| 10. Limit to English language |

**PsycInfo Search Strategy**

| 1. exp Home Care/ or exp Caregivers/ or carer.mp.  **OR**  2. ("informal care*" or "famil* care*" or "spous* care*" or "unpaid care*").mp. [mp=title, abstract, heading word, table of contents, key concepts, original title, tests & measures, mesh] |
| --- |
| **AND** |
| 3. support group.mp. or exp Support Groups/  **OR**  4. exp Group Counseling/ or exp Group Psychotherapy/ or exp Group Intervention/ or therapy group.mp.  **OR**  5. exp Self-Help Techniques/ or self-help group.mp.  **OR**  6. ("group based" adj4 intervention).mp. [mp=title, abstract, heading word, table of contents, key concepts, original title, tests & measures, mesh]  **OR**  7. ("group based" adj4 program*).mp. [mp=title, abstract, heading word, table of contents, key concepts, original title, tests & measures, mesh] |
| **AND** |
| 8. exp Presenile Dementia/ or exp Dementia/ or exp Senile Dementia/ or exp Semantic Dementia/ or dementia.mp. or exp Dementia with Lewy Bodies/ or exp Vascular Dementia  **OR**  9. alzheimer's disease.mp. or exp Alzheimer's Disease/ |
| **AND** |
| 10. Limit to English language |

**Web of Science Search Strategy**

| TOPIC: (carer OR caregiver OR "informal care*" or "famil* care*" or "spous* care*" or "unpaid care*") |
| --- |
| **AND** |
| TOPIC: ("support group" or "therapy group" or "self-help group" or "group counseling" or ("group based" NEAR/4 intervention) or ("group based" NEAR/4 program)) |
| **AND** |
| TOPIC: (dementia OR alzheimer's disease) |

**Online Supplementary Material Section B**

*Data extraction table (including quality assessment).*

Extraction table for the following topics: (1) Author(s) and location and publication year (2) Journal of publication (3) Study design (4) Type of intervention (5) Characteristics of intervention (6) Participant demographics (7) Type of data collected (8) Key findings (9) Quality of evidence. The quality of included studies was assessed using standardised Critical Appraisal Skills Programme (CASP) checklists for qualitative and randomised control trial (RCT) design studies (CASP, 2017). The Joanna Briggs Institute (JBI) Critical Appraisal checklist for quasi-experimental studies was used for non-randomised quantitative studies and the Mixed-Methods Appraisal Tool (MMAT) checklist was used to evaluate mixed methods research (Hong et al., 2018).

| (1) Author, location, and year | (2) Journal of publication | (3) Study Design | (4) Type of intervention | (5) Characteristics of intervention | (6) Participant demographics | (7) Type of data collected | (8) Key findings | (9) Quality of evidence |
| --- | --- | --- | --- | --- | --- | --- | --- | --- |
| Subcategory 1: Group CBT interventions (5 studies) | | | | | | | | |
| Aboulafia-Brakha et al  Switzerland  2014 | Aging & Mental Health | Quantitative  RCT (2 groups) | Group CBT and Psycho-educational (control group) | Duration: 8 weekly sessions  Carers of people with moderate to severe Alzheimer’s disease were randomly assigned to a CBT group or psychoeducational group. Interventions were delivered face-to-face in a group setting. The CBT group were divided into 6-8 people for 90-minute sessions, the psychoeducational group sessions were 60 minutes and conducted for all 17 participants together. | N = 35  **Intervention group  (n = 17)**  Gender - 100% Female  Mean Age - 59.42  Familial Relationship - Spouse 75%; Parent 16.70%  **Control group  (n = 18)**  Gender –67% Female  Mean Age –55.07  Familial Relationship - Spouse 80%; Parent 13.30% | Primary outcome measure: Salivary cortisol levels  Secondary outcome measures (questionnaire-based psychometric tests): stress, burden, depression, trait anxiety  For care recipient: function in daily life, neuropsychiatric symptoms.  Data was collected before and after the intervention. | Salivary cortisol levels significantly decreased after intervention in the CBT group only. Both groups showed a reduction of neuropsychiatric symptoms of their care recipient after intervention but no significant change in the well-being of the carer. | Low |
| Arango-Lasprilla et al  Colombia  2014 | American Journal of Alzheimer's Disease and Other Dementias | Quantitative  RCT (2 groups) | Group CBT and Psycho-educational (control group) | Duration: 8 weekly sessions  69 carers of people with dementia were randomly assigned to a CBT group therapy or an educational control condition. Both types of session were 2-hours long and were weekly and were conducted in groups of 6-10 people. | N = 69  **Intervention group  (n = 39)** Gender – 87.2% Female  Mean Age – 59.4  Familial Relationship –Spouse 17.9% Parent 56.4% Sibling 12.8% Other 13.8%  **Control group  (n = 30)**  Gender – 73.3% Female  Mean Age – 55.1  Familial Relationship - Spouse 20% Parent 46.7% Sibling 10% Other 23% | A battery of questionnaires was administered pre-intervention, post-intervention, and 3 months later as a follow up.  The questionnaires measured: depression, burden, life satisfaction and stress. | The CBT group had higher life satisfaction and lower levels of depression and burden than the control group post-intervention and at follow up.  There was no improvement of stress levels in either group. | Moderate |
| Gendron et al  Canada  1996 | Clinical Gerontol-ogist | Quantitative  RCT (2 groups) | Group CBT and Psycho-educational (control group) | Duration: 8 weekly sessions  35 spousal carers of people with dementia were recruited. Participants were randomly assigned to a CBT group therapy intervention or a information group which acted as a control group. The information group watched an informational video about dementia and then had a question-and-answer session. Both types of session were 90 minutes in length. | N = 35  (intervention group n = 18; control group n = 17)  Gender – 65.7% Female  Mean Age – 66.2  Familial Relationship –not reported | Questionnaires (mental health, negative thinking, burden, assertiveness) were administered pre-intervention and post-intervention. 26 participants were interviewed as a follow up 3 to 6 months after the intervention. | Participants in the CBT group had higher assertiveness post-intervention (e.g. help seeking behaviour, assertive in asking for help from other family members). There were no significant improvements in either group for any other measure.  There was no difference between the groups at the follow up interviews. | Low |
| Gonyea et al  USA  2006 | Gerontol-ogist | Quantitative  RCT (2 groups) | Group CBT and Psycho-educational (control group) | Duration: 5 weekly sessions  Carers of people with dementia were randomly assigned to a group CBT condition or a psycho-educational control group. The interventions were delivered in groups of 5-10 people over 5 weekly sessions that lasted 90-minutes. | N = 80 (intervention group n = 40; control group n = 40)  Gender – 67% Female  Mean Age – 64.4  Familial Relationship –Spouse 59% Parent 32% Other 9% | Structured interview to assess burden and a questionnaire to assess neuropsychiatric symptoms in the care recipient (and the level of distress experienced by the carer as a result of those symptoms) were conducted pre-intervention and post-intervention. | Participants in the CBT group reported significantly lower levels of distress in response to neuropsychiatric symptoms post-intervention compared to the control group.  Burden did not significantly decrease in either group. | Moderate |
| Passoni et al  Italy  2014 | Alzheimer Disease & Associated Disorders | Quantitative  Quasi-experimental (3 groups) | Group CBT or Self-help manual (or treatment as usual control group) | Duration: 6 biweekly sessions  Carers of people with dementia were assigned to a CBT group therapy condition, a self-help manual condition or a control group that received usual care. CBT sessions were 2 hours and contained 7-10 people. The manual contained information about dementia and caring. | N = 102 (CBT group n = 39, manual group n = 30, control group n = 33)  Gender – 61.7% Female  Mean Age – 58.5  Familial Relationship –not reported | Questionnaires (depression, anxiety, carer needs) were administered pre-intervention and 6 months from the start of the intervention. | Both the CBT group and the manual group showed a significant reduction in their care needs post-intervention compared to the control group. Though. there was no significant difference between the reduction seen in the CBT group and the manual group.  The CBT and manual interventions did not have a caregiver significant effect on anxiety or depression. | Moderate |
| Subcategory 2: Group Psycho-educational Interventions (8 studies) | | | | | | | | |
| Andren & Elmstahl  Sweden  2008 | Nordic College of Caring Science | Quantitative  Quasi-experimental (2 groups) | Psycho-educational programme followed by support group | Duration: 5 weekly sessions  Followed by  biweekly support group sessions for 3 months  Carers of people with dementia attended 5 weekly group-based psychoeducational sessions that lasted two hours. After these were completed, participants then attended a biweekly 90-minute support group for 3 months aimed at providing emotional support. This group was compared to a control group who had no intervention from a similar, nearby district. | N = 308  **Intervention group  (n = 153)**  Gender – 61% Female  Mean Age – 62  Familial Relationship – Spouse 25%  Parent 60%  Other 15%  **Control group (n = 155)**  Gender – 68% Female  Mean Age – 62  Familial Relationship – Spouse 21%  Parent 65%  Other 14% | A structured interview was conducted prior the intervention and subsequently a telephone based structured interview was conducted every 3 months.  Questionnaires were issued 6 months and 12 months after the start of the intervention measuring burden, life satisfaction and level of disability in the care recipient. | Carers in the intervention group reported significantly lower strain and disappointment in the 6 and 12-month follow up questionnaires than the control group. However, there was no change in all other questions of the burden questionnaire.  Life satisfaction increased in the intervention group and decreased in the control group. | Moderate |
| Hsu et al  Taiwan  2017 | Alzheimer's and Dementia | Quantitative  Pretest-posttest design (no control group) | Psycho-educational intervention | Duration: 4 weekly workshops (8 hours long)  Carers of people with dementia completed an intensive 32-hour psychoeducational training course aimed at decreasing burden and improving mental and physical health. | N = 53  Gender – 85.3% Female  Mean Age – 52.5  Familial Relationship – 29% Spouse  62% Parent  9% Parent-in-law | Questionnaires (burden, health) were conducted pre-intervention and then at 3 months, 6 months and 9 months post-intervention. | Only 34 out of the 53 participants completed the intervention and assessments. There was a significant improvement in burden and mental health but there was no improvement in physical health. | Low |
| Küçükgüçlü et al  Turkey  2018 | Geriatric Nursing | Mixed methods  Pretest-posttest design (no control group) | Psycho-educational intervention | Duration: 10 sessions  37 carers of people with dementia attended 10 sessions of a psychoeducational group intervention held every other week. Sessions were two hours and the size of the groups were 18 people and 19 people. | N = 30  Gender – 100% Female  Mean Age – 58.3  Familial Relationship – 40% Spouse  62% Parent | Questionnaire to measure burden administered pre-intervention and post-intervention.  Semi-structured focus group interviews were conducted post-intervention. | There was a significant decrease in burden post-intervention (p = 0.49, therefore it was only just able to reach significance).  Three themes emerged from the interviews:  1. Having knowledge (gaining a better understanding of their situation and how to manage it)  2. Calming down (having the opportunity to release unexpressed emotions and make friends)  3. Acceptance (accepting that dementia is progressive and accepting what the future will be like) | Moderate |
| Kurz et al  Germany  Austria  Switzerland  2010 | International Journal of Geriatric Psychiatry | Quantitative  RCT (2 groups) | Psycho-educational intervention | Duration: 7 biweekly sessions  A multicentre randomised control trial including 292 carers of people with dementia.  Participants in the intervention group attended 7 90-minute biweekly educational group sessions aimed at education and problem solving. Following this, 6 bimonthly refresher meetings were held.  Participants in the control group had one individual counselling session with a social worker and were directed to services in case of emergency. | N = 292  **Intervention group  (n = 156)**  Gender – 71.8% Female  Mean Age – 62.6  Familial Relationship – 60% Spouse  38% Parent  **Control group (n = 136)**  Gender – 66.2% Female  Mean Age – 62  Familial Relationship – 55% Spouse  40% Parent | Quality of life and depression measures were administered by a blinded researcher pre-intervention and post-intervention. | The intervention had no effect on depression or quality of life and did not impact nursing home admission of the care recipient.  Participants in the intervention group were significantly more satisfied with the intervention than the control group and said the intervention was helpful (130 participants in the intervention group compared to 118 in the control group). | Moderate |
| Lewis et al  USA  2009 | Rehabilitation Nursing Journal | Quantitative  Pretest-posttest design (no control group) | Psycho-educational intervention | Duration: 9 weekly sessions  Carers of people with dementia participated in 90 minutes long psychoeducational support group sessions with 30 minutes of relaxation techniques weekly for 9 weeks. | N = 209  Gender – not reported  Mean Age – 52.8  Familial Relationship – not reported | Questionnaires (burden, quality of life, stress, depression, anxiety, anger) were administered pre-intervention, halfway through the intervention (after 4 weeks), post-intervention and two months after the completion of the intervention. | There was a significant improvement post-intervention and at 2-month follow up in all measures. | Moderate |
| Martin-Carrasco et al  Spain  2014 | Alzheimer Disease and Associated Disorders | Quantitative  RCT (2 groups) | Psycho-educational intervention | Duration: 7 biweekly sessions  Carers of people with dementia were randomly assigned a psycho-educational intervention group or a control group that received standard care. The intervention was administered over 7 biweekly sessions which lasted 90 to 120 minutes. | N = 238  **Intervention group  (n = 115)**  Gender –77.4% Female  Mean Age – 61  Familial Relationship – 47.8% Spouse  47% Parent  5.2% Other  **Control group (n = 123)**  Gender –77.2% Female  Mean Age – 63.2  Familial Relationship – 50.4% Spouse  43.9% Parent  5.7% Other | Questionnaires (burden, distress, health, quality of life) were administered pre-intervention and as a 4-month follow up. | There was a significant decrease in anxiety and insomnia items on the health measure in the intervention group post-intervention. However, there was no significant improvement in any other measure. The authors conclude that the intervention is not better than standard care. | Moderate |
| Pihet & Kipfer  Switzerland  2018 | BMC Geriatrics | Mixed methods  Pretest-posttest design (no control group) | Psycho-educational intervention | Duration: 15 weekly sessions  Carers of people with dementia participated in 15 weekly 2-hour long psycho-educational group sessions. | N = 19  Gender –73% Female  Mean Age – 68  Familial Relationship – 69% Spouse  27% Parent  7% Siblings | Qualitative semi-structured interviews were conducted pre-intervention and post-intervention. A battery of questionnaires (burden, distress, self-efficacy) was also taken at this time.  Twice a week throughout the intervention participants answered short questions about daily life through a tablet app. | Three themes emerged from the qualitative interviews:  1. Sharing experiences and strategies (participants were able to learn from each other which strengthened their education).  2. Being in the same boat (feeling understood and less alone).  3. Being able to cope (participants reported that the intervention gave them strategies that helped they cope and manage challenging issues)  Quantitative data showed that there was a significant improvement of burden and self-efficacy. The carers psychological distress also improved significantly but their distress in response to the psychiatric symptoms of the care recipient did not improve.  The tablet questions revealed that carers changed their caring strategies in daily life over the course of the intervention. | Moderate |
| Ulstein et al  Norway  2007 | Dementia and Geriatric Cognitive Disorders | Quantitative  RCT (2 groups) | Psycho-educational intervention | Duration: 6 weekly sessions  In this multi-centre randomised control trial, carers of people with dementia attended a 3-hour lecture about dementia and its symptoms and then attended a weekly 2-hour psychoeducational support group for 6 weeks. The carers in the control group received treatment as usual. | N = 180  (intervention group n = 90; control group n = 90)  Gender – n/a  Mean Age – not reported  Familial Relationship – n/a | Questionnaires were conducted pre-intervention, post-intervention and 12 months after baseline. The outcome measure for carers was stress. Data was also collected for the care recipient about neuropsychiatric symptoms, MMSE, activities of daily living and disability. | No significant effect on any outcome measure (for both carers and care recipients) | Moderate |
| Subcategory 3: Support Group Interventions (6 studies) | | | | | | | | |
| Acton & Miller  USA  1996 | Issues in Mental Health Nursing | Mixed Methods  Pretest-posttest design (no control group) | Support group | Duration: Biweekly sessions for 12 months  Carers of people with Alzheimer’s disease were recruited to take part in a support group for 6-7 people aimed at improving carer hardiness and resistance to stress. | N = 26  Gender –77% Female  Mean Age – 75  Familial Relationship – 54% Spouse  46% Parent | Semi-structured interviews and questionnaires were conducted prior to intervention and then 6-months and 12-months into the intervention.  Questionnaires:  Demographic data, quality of life, stress  1-2 months after the intervention ended 14 participants were interviewed about their experiences. | Four themes emerged in the interviews about why the support group was an effective intervention:  1. Affiliation (feeling connected to others)  2. Individuation (improvement to the sense of self and gaining a sense of control)  3. Self-acceptance (feeling better about themselves)  4.Healing (gaining a feeling of peace)  Follow up interviews revealed that caregivers were able to maintain and increase their ability to cope with caring.  No effect on quality of life or stress was found in the quantitative data. | Moderate |
| Berger et al  Germany  2004 | International Journal of Geriatric Psychiatry | Quantitative  Quasi-experimental | Support group | Duration: Weekly sessions for 2 years  Participants were carers of people with dementia recruited from a memory clinic. Participants in the intervention group attended weekly 1 hour support group sessions over 2 years and were compared to demographically matched controls. Whilst participants attended the support group their care recipient attended a music therapy group, but these findings will not be discussed in the present work.  Participants in the control group received treatment as usual. | N = 36  **Intervention group  (n = 18)**  Gender –66.6% Female  Mean Age – 64  Familial Relationship – 83.3% Spouse  11% Parent  5.5% Other  **Control group (n = 18)**  Gender –61.1% Female  Mean Age – 60.1  Familial Relationship – 72.2% Spouse  27.7% Parent | Questionnaires on depression and burden were completed pre-intervention and at 6, 12 and 24 months into the intervention. | No effect on caregiver burden or depression at any point in the study. | Moderate |
| Chu et al  Taiwan  2011 | Journal of Aging and Health | Quantitative  RCT (2 groups) | Support group | Duration: 12 weekly sessions  Carers of people with dementia took part in a 12-week support group intervention. The control group received treatment as usual. | N = 60  **Intervention group (n = 30)**  Gender –53.3% Female  Mean Age – not reported  Familial Relationship – not reported  **Control group (n = 30)**  Gender –60% Female  Mean Age – not reported  Familial Relationship – not reported | Questionnaires (depression and burden) were administered pre-intervention, post-intervention and as a 1 month follow up after the intervention had finished. | There was no significant change to depression or burden. | Moderate |
| Fung & Chien  Hong Kong  2002 | Archives of Psychiatric Nursing | Quantitative  RCT (2 groups) | Support group | Duration: 12 weekly sessions  Fifty-two family carers of people with dementia from two Hong Kong health centres were recruited to participate. Participants in the intervention group attended 12 weekly hour-long sessions of a mutual support group. The control group had treatment as usual and did not receive any group-based support in this period. | N = 52  **Intervention group (n = 26)** Gender –61.5% Female  Mean Age – n/a  Familial Relationship – 46.2% Spouse  **Control group (n = 26)** Gender –65.4% Female  Mean Age – n/a  Familial Relationship – 53.8% Spouse | Questionnaires (distress and quality of life) were administered pre-intervention and post-intervention. | The intervention group had a significant improvement to distress and quality of life post-intervention compared to the control group. | Moderate |
| Lauritzen et al  Denmark  2019 | Journal of advanced nursing | Qualitative  Ethnography | Support group | Duration: 6-9 weekly meetings  Semi-structured interviews were conducted with 25 carers of people with dementia who attended a support group. Additional data was gathered through observation of four support groups. | N = 25  Gender –76% Female  Age – 40-83 (range)  Familial Relationship – not reported | n/a | Three themes emerged from the interviews and from the observation field notes:  1. Emotional well‐being due to peer and family support (positive interactions with others lifted the carer’s moods and allowed them to express their emotions and hear other’s perspectives on similar problems)  2. Emotional sense of togetherness despite hardships (connecting with others in similar situations raised carers self-esteem and confidence)  3. Emotional and ethical considerations in caregiving (gaining an understanding of how to treat the caregiver with respect and dignity as their condition progresses and ensure that they receive the same treatment from others) | Low |
| Winter & Gitlin  USA  2006 | American Journal of Alzheimer's Disease & Other Dementias | Quantitative  RCT (2 groups) | Support group | Duration: Weekly sessions for 6 months  Female carers of people with dementia took part in a telephone-based support group. There were 5 carers in each support group, and they talked for 1-hour weekly for 6 months. The control group received treatment as usual. | N = 103  **Intervention group  (n = 58)**  Gender –100% Female  Mean Age – 68.7  Familial Relationship – 34.5% Spouse  **Control group (n = 45)**  Gender –100% Female  Mean Age – 64  Familial Relationship – 46.9% Spouse | Structured interviews (depression, burden, personal gains) were conducted via telephone pre-intervention and then 6-months later. | No significant effect on any outcome measure.0 | Moderate |

**Online Supplementary Material Section C**

*Summary of the content included in each of the intervention sessions in the group cognitive behavioural therapy sub-category.*

| **Session** | **Aboulafia-Brakha et al. (2014)** | **Arango-Lasprilla et al. (2014)** | **Gendron et al. (1996)** | **Gonyea et al. (2006)** | **Passoni et al. (2014)** |
| --- | --- | --- | --- | --- | --- |
| **1** | Group introduction | Explanation of CBT. Discussion of feelings of frustration and anger about being a carer. Relaxation skills and anger management. | A session-by-session breakdown was not included.  Coping skills training included:   - Assertion training for dealing with family members and health services etc. - Problem solving training to overcome challenging situations - Cognitive restructuring to tackle negative thought patterns - Discussion of caring experiences | Introduction: Overview of Group Goals and Guidelines | Understanding the stages of Dementia, and Discussion of Caregivers’ Problems |
| **2** | Life changes brought on by becoming a carer |  |  | Increasing Pleasant Events and Improving Communication | Advice for the Management of Everyday Life |
| **3** | Implicating other relatives in caregiving; engaging in leisure activities | Practicing and developing cognitive techniques for coping with frustration and changing dysfunctional thoughts |  | Increasing Pleasant Events and Understanding Behaviour | Self-care and techniques for avoiding catastrophic thoughts |
| **4** | Understanding and dealing with cognitive symptoms |  |  | Understanding and Changing Difficult Behaviours | Understanding Behavioural and Psychological Symptoms of Dementia |
| **5** | Understanding and dealing with behavioural manifestations |  |  | Review of the programme | The Management of the Home Environment |
| **6** | Re-establishing bonds with the care recipient | Developing assertiveness skills and continuing relaxation exercises |  |  | Signposting to support |
| **7** | Anticipatory grief and disease progression |  |  |  |  |
| **8** | Recall of session topics and feedback | Final review of the programme |  |  |  |

*Note.* Grey cells indicate that no session took place as the intervention was shorter than 8 weeks in duration.

**Online Supplementary Material Section D**

*Summary of the content included in each of the intervention sessions in the psycho-educational sub-category.*

| **Session** | **Andren & Elmstahl (2008)**  **Sweden** | **Hsu et al. (2017)**  **Taiwan** | **Küçükgüçlü et al. (2018)**  **Turkey** | **Kurz et al. (2010)**  **Germany**  **Austria**  **Switzerland** |
| --- | --- | --- | --- | --- |
| 1 | Introduction to the programme. Direct teaching on dementia disease followed by identiﬁcation and discussion of some of the problems and difﬁculties experienced by family caregivers. | A session-by-session breakdown was not included but the content of the 4-week intervention was as follows:   - General information about dementia. - Information about medical and welfare services for persons with dementia. - Improving communication skills and developing strategies for managing challenging behaviour. | Introduction to the programme. Participants are taught general information about dementia. | Introduction to Alzheimer’s disease |
| 2 | Teaching on depression, confusion, and dementia diagnosis. |  | Discussion of home care techniques. | Discussion of caregiving skills needed for the early stages of Alzheimer’s disease. |
| 3 | Resources and services available from the community services. Planning for the future. |  | Discussion of medication management | Discussion of caregiving skills needed for the moderate stage of Alzheimer’s disease. |
| 4 | Communication with people with dementia. Promoting positive attitudes towards people with dementia. Coping with challenging forms of behaviour. |  | Communication with people with dementia. | Discussion of caregiving skills needed for the later stage of Alzheimer’s disease. |
| 5 | Interaction and establishment of contact with people with dementia. Developing new skills and knowledge. Self-care of the caregiver. |  | Self-care of the caregiver. | Help with legal and insurance related issues e.g. power of attorney. |
| 6 |  |  | Planning for future care needs and information about formal services that carers can access for support. | Signposting to support. |
| 7 |  |  | Discussion of the difficulties of the caregiving process and developing coping methods. | General discussion of personal problems (no predetermined content for this session). |
| 8 |  |  |  |  |
| 9 |  |  |  |  |
| 10 |  |  | Evaluation of the intervention through focus group interviews. |  |
| **Session** | **Lewis et al. (2009)**  **USA** | **Martin-Carrasco et al. (2014)**  **Spain** | **Pihet & Kipfer (2018)**  **Switzerland** | **Ulstein et al. (2007)**  **Norway** |
| 1 | Relaxation techniques for managing the stress of caregiving, including:   - Breathing techniques - Meditation - Muscle relaxation - Guided imagery | Strain and well-being. | A session-by-session breakdown was not included but the content of the 15-week intervention focused on the identification of stressful and challenging aspects of caring for someone with dementia and developing coping strategies in response to these situations.  The program used a combination of:   - Teaching and providing information about dementia - Group discussions - Work on personal stressful situations - Exercises to be completed at home | 3-hour lecture about the symptoms and typical course of dementia. Participants were also taught about pharmacological and non-pharmacological treatments for dementia. |
| 2 |  | Strategies for managing challenging behaviour. |  | After the first session the participants took part in 6 2-hour group meetings focused on communication and problem-solving techniques. |
| 3 | Discussion of grief and loss as a result of dementia and using art therapy to explore this. Discussion of how to manage depression. |  |  |  |
| 4 | Discussion of self- and environment-focused coping strategies. Introduction to aromatherapy as a relaxation technique. | Addressing negative thoughts. |  |  |
| 5 | Understanding and managing difficult and challenging symptoms of dementia. Introduction to massage as a relaxation technique. | Ways to communicate with people with dementia. |  |  |
| 6 | Positive thinking and journaling. | Planning for the future. |  |  |
| 7 | Self-care of the caregiver and music therapy. | Planning enjoyable activities. |  |  |
| 8 | Overview of the previous seven sessions and discussion of how the participants will use the techniques they have learned in the future. |  |  |  |
| 9-15 |  |  |  |  |

*Note.* Grey cells indicate that no session took place as the intervention was shorter than 10-15 weeks in duration.
